# Supplementary material for: The Systems Biology Research Tool: evolvable open-source software
Source: BMC Syst Biol. 2008 Jun 29;2:55. doi: 10.1186/1752-0509-2-55 (PMC2446383; doi:10.1186/1752-0509-2-55)
Supplement: Additional file 1 — SBRT Archive. An archive of the current version of the Systems Biology Research Tool. [file 1752-0509-2-55-S1.zip › sbrt-1.4.0/doc/developers_guide/api/sbrt/kernel/math/algebra/AbstractSmdMatrix.html]

AbstractSmdMatrix


|  |  |  |
| --- | --- | --- |
| **Overview** \| **Package** \| **Class** \| **Use** \| **Tree** \| **Deprecated** \| **Index** \| **Help** | |  |
| **PREV CLASS**   **NEXT CLASS** | **FRAMES**    **NO FRAMES**     **All Classes** |  |
| SUMMARY: NESTED \| FIELD \| CONSTR \| METHOD | DETAIL: FIELD \| CONSTR \| METHOD |  |


---


## sbrt.kernel.math.algebra Class AbstractSmdMatrix<R,C>

```
java.lang.Object
  sbrt.kernel.math.algebra.AbstractMdMatrix<R,C>
      sbrt.kernel.math.algebra.AbstractSmdMatrix<R,C>
```

**Type Parameters:**: `R` - the row type.: `C` - the column type.

**All Implemented Interfaces:**: DoubleMatrix<R,C>, DoubleMatrixBuilder<R,C>, Matrix<R,C,java.lang.Double>, MatrixBuilder<R,C,java.lang.Double>, SmdMatrix<R,C>, SparseDoubleMatrix<R,C>

**Direct Known Subclasses:**: HashMatrix, RowOrTreeMatrix, TreeMatrix

---

``` public abstract class AbstractSmdMatrix<R,C> extends AbstractMdMatrix<R,C> implements SmdMatrix<R,C> ```

This abstract class is a skeleton implementation of
`SmdMatrix`.

**Author:**
:   This class was written and documented by
    Jeremiah Wright while in the Wagner lab.

---

**Constructor Summary**

`AbstractSmdMatrix()`


| **Method Summary** | |
| --- | --- |
| `boolean` | `equals(java.lang.Object obj)`             Indicates if the provided object is equal to this matrix. |
| `<X extends R,Y extends C> void` | `setElement(X rowIndex, Y columnIndex, double value)`             Sets the element at the provided row and column indices to the provided double precision value. |
| `void` | `setElements(java.util.Map<IndexPair<R,C>,java.lang.Double> elements)`             Sets the corresponding elements of this matrix to those provided. |
| `void` | `setElements(Matrix<? extends R,? extends C,? extends java.lang.Double> m)`             Sets the corresponding elements of this matrix to those of the provided matrix. |
| `void` | `setElements(SparseDoubleMatrix<? extends R,? extends C> m)`             Sets the corresponding elements of this matrix to those of the provided matrix. |
| `java.util.List<LinearComb<C>>` | `toLinearCombs()`             Returns this matrix as a list of linear combinations. |

**Methods inherited from class sbrt.kernel.math.algebra.AbstractMdMatrix**

`isEmpty, setElements, toString`

**Methods inherited from class java.lang.Object**

`clone, finalize, getClass, hashCode, notify, notifyAll, wait, wait, wait`

**Methods inherited from interface sbrt.kernel.math.algebra.DoubleMatrixBuilder**

`setElements`

**Methods inherited from interface sbrt.kernel.math.algebra.MatrixBuilder**

`removeColumn, removeRow, setElement`

**Methods inherited from interface sbrt.kernel.math.algebra.Matrix**

`columns, getColumnIndices, getRowIndices, hashCode, isColumnIndex, isEmpty, isRowIndex, rows, toString`

**Methods inherited from interface sbrt.kernel.math.algebra.DoubleMatrix**

`getElement`

**Methods inherited from interface sbrt.kernel.math.algebra.Matrix**

`columns, getColumnIndices, getRowIndices, hashCode, isColumnIndex, isEmpty, isRowIndex, rows, toString`

**Methods inherited from interface sbrt.kernel.math.algebra.SparseDoubleMatrix**

`getColIndNzEl, getColumn, getRow, getRowIndNzEl`

**Methods inherited from interface sbrt.kernel.math.algebra.DoubleMatrix**

`getElement`

**Methods inherited from interface sbrt.kernel.math.algebra.Matrix**

`columns, getColumnIndices, getRowIndices, hashCode, isColumnIndex, isEmpty, isRowIndex, rows, toString`

**Constructor Detail**

### AbstractSmdMatrix

```
public AbstractSmdMatrix()
```


**Method Detail**

### setElement

```
public <X extends R,Y extends C> void setElement(X rowIndex,
                                                 Y columnIndex,
                                                 double value)
```

:   Sets the element at the provided row and column indices
    to the provided double precision value.

    :   **Specified by:**: `setElement` in interface `DoubleMatrixBuilder<R,C>`
    :   **Type Parameters:**: `X` - a type extending the primary row type.: `Y` - a type extending the primary column type. **Parameters:**: `rowIndex` - the row index.: `columnIndex` - the column index.: `value` - the double precision element value.

---


### setElements

```
public void setElements(Matrix<? extends R,? extends C,? extends java.lang.Double> m)
```

:   Sets the corresponding elements of this matrix to
    those of the provided matrix.

    :   **Specified by:**: `setElements` in interface `MatrixBuilder<R,C,java.lang.Double>`
    :   **Parameters:**: `m` - the matrix whose elements are to be copied into this matrix.

---


### setElements

```
public void setElements(SparseDoubleMatrix<? extends R,? extends C> m)
```

:   Sets the corresponding elements of this matrix to
    those of the provided matrix.

    :   **Specified by:**: `setElements` in interface `SmdMatrix<R,C>`
    :   **Parameters:**: `m` - the matrix whose elements are to be copied into this matrix.

---


### setElements

```
public void setElements(java.util.Map<IndexPair<R,C>,java.lang.Double> elements)
```

:   Sets the corresponding elements of this matrix to
    those provided.

    :   **Parameters:**: `elements` - the elements to be copied into this matrix.

---


### equals

```
public boolean equals(java.lang.Object obj)
```

:   Indicates if the provided object is equal to this matrix.
    Two matrices are considered equal if they share the same
    sets of column and row indices and the elements in each
    matrix for each column-row index pair are the same.

    :   **Specified by:**: `equals` in interface `Matrix<R,C,java.lang.Double>` **Overrides:**: `equals` in class `AbstractMdMatrix<R,C>`
    :   **Parameters:**: `obj` - the object with which to compare. **Returns:**: `true` if this object equals the provided object; `false` otherwise.

---


### toLinearCombs

```
public java.util.List<LinearComb<C>> toLinearCombs()
```

:   Returns this matrix as a list of linear combinations. In other
    words, this method returns the product of this matrix with a
    column vector of the column indices of this matrix.

    :   **Specified by:**: `toLinearCombs` in interface `DoubleMatrix<R,C>`
    :   **Returns:**: this matrix as a list of linear combinations.


---


|  |  |  |
| --- | --- | --- |
| **Overview** \| **Package** \| **Class** \| **Use** \| **Tree** \| **Deprecated** \| **Index** \| **Help** | |  |
| **PREV CLASS**   **NEXT CLASS** | **FRAMES**    **NO FRAMES**     **All Classes** |  |
| SUMMARY: NESTED \| FIELD \| CONSTR \| METHOD | DETAIL: FIELD \| CONSTR \| METHOD |  |


---
